# Experiences and facilitators of physical activity engagement amongst colorectal and endometrial cancer survivors: the Wearable Activity Technology and Action-Planning (WATAAP) trial

**DOI:** 10.1007/s00520-023-08137-z

**Published:** 2023-11-02

**Authors:** Sarah J. Hardcastle, Emma Douglass, Bree Wilson, Chloe Maxwell-Smith

**Affiliations:** 1 Department of Sport and Physical Activity, Sheffield Hallam University, Broomhall, Sheffield, UK; 2 Institute for Health Research, University of Notre Dame Australia, Fremantle, Western Australia Australia; 3 School of Population Health, Curtin University, Perth, Western Australia Australia; 4 Optimising Health and Wellbeing Domain, Enable Institute, Curtin University, Perth, Western Australia Australia

**Keywords:** Adherence, Behaviour change, Cancer survivors, Exercise, Oncology

## Abstract

**Purpose:**

This study explored colorectal and endometrial cancer survivors’ experiences of participation in a wearable intervention and the dimensions that influenced intervention engagement and physical activity behaviour change.

**Methods:**

Semi-structured interviews (*n*= 23) were conducted with intervention participants (mean age 65.8 (SD ±7.1) and analysed using thematic analysis.

**Results:**

Four main themes were identified: (i) commitment, (ii) accountability and monitoring, (iii) routine, (iv) Fitbit as health coach. Those that assigned a higher priority to PA were more likely to schedule PA and be successful in PA change. Those less successful presented more barriers to change and engaged in more incidental PA. The Fitbit acting as health coach was the active ingredient of the intervention.

**Conclusions:**

Commitment evidenced through prioritising PA was the foundational dimension that influenced PA engagement. Interventions that foster commitment to PA through increasing the value and importance of PA would be worthwhile. Wearables holds great promise in PA promotion and harnessing the technique of discrepancy between behaviour and goals is likely a valuable behaviour change technique.

**Supplementary Information:**

The online version contains supplementary material available at 10.1007/s00520-023-08137-z.

## Background

In 2020, ~19.3 million new cancers were recorded worldwide and almost 10 million deaths [1]. In relation to the population in the present study, colorectal cancer (CRC) represents the third most diagnosed cancer (10%) and the second leading cause of cancer death (9.4%), and endometrial cancer is the tenth most diagnosed cancer and comprises 3% of all cancer cases globally [1].

Increased physical activity (PA) post-diagnosis has been demonstrated to reduce both cancer-specific mortality and all-cause mortality in both CRC [2, 3] and endometrial cancer survivors [4]. Despite the increasing evidence that PA improves cancer outcomes, most survivors [5] fail to meet the aerobic guidelines of at least 150 min of moderate-intensity-PA/per week [6].

Despite the evidence supporting the benefits of PA for cancer survivors, most studies have focused on group differences, whilst giving little attention to individual differences or to understanding the ‘active ingredients’ of interventions. To improve our understanding of the efficacious components of interventions and understand the broader dimensions associated with PA behaviour change (PABC), qualitative approaches are worthwhile [7].

Qualitative approaches capture the range of influences on behaviour and offer an in-depth perspective on individuals’ perceptions and experiences that may help to identify the salient dimensions that influence intervention engagement and PABC.

There is a relative dearth of research concerning the experiences of cancer survivors following participation in a PA intervention [8–10]. Grimmett et al. (2020) found that enjoyable and highly valued PA was associated with PA maintenance following intervention [8]. Low motivation and a lack of enjoyment typified those who were insufficiently active [8]. Midtgaard et al. (2012) [10] found that goal-setting and prioritising PA typified exercise maintenance. Kokts-Porietis et al. (2019) [9] found the barriers of time, lack of motivation and bad weather affected adherence to a home-based intervention in breast cancer survivors. Feedback via the wearable-tracker was a facilitator of PA adherence. However, wearables were experienced by some as a source of judgement and failure when tracker-feedback did not correspond to perceptions of PA achievement [9].

The present study originates from the WATAAP (Wearable Activity Technology and Action-Planning) intervention [11] to ascertain whether Fitbits, in conjunction with action-planning, and was effective in increasing moderate-vigorous PA (MVPA) in endometrial and CRC survivors. The WATAAP intervention produced a significant increase in MVPA [11] that was maintained at follow-up [12]. The aim of the present study was to identify the active ingredients and salient dimensions that influenced PABC and intervention engagement amongst intervention participants.

## Methods

### Participants

Eligible participants were cancer survivors who participated in the WATAAP intervention [13]. The full eligibility criteria and intervention have been described previously [11, 13]. The 12-week intervention consisted of three components: (i) the provision of a Fitbit Alta^TM^; (ii) two 2-h group sessions to include PA recommendations, goal-setting, action-planning, coping-planning and self-monitoring; and (iii) a 20-min phone call (week 8) to provide support and assist with coping-planning.

### Procedure

The current study conformed to the Standards for Reporting Qualitative Research [14] (see online Supplemental File 1). The St. John of God Hospital Ethics Committee approved this study (#1102). Participants indicated their willingness to participate in an interview to discuss their experiences of the intervention at their final assessment. A research assistant (RA) contacted participants who expressed willingness and an interview date was arranged. Participants provided written, informed consent prior to the interview and were informed that pseudonyms would be used in any reporting of the data.

**Fig. 1:**
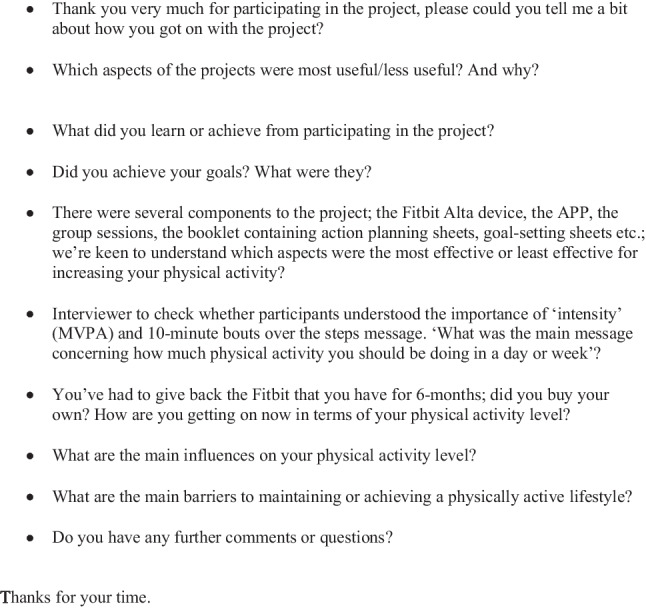
Interview guide

Semi-structured interviews (mean duration = 1.25 h) in May/June 2018 were conducted by two RAs (final year Psychology students) not involved in intervention delivery. The RAs were trained by the lead author (SH) who has a wealth of experience in qualitative data collection and analysis, and in PABC. Interviews took place within 3 months of trial completion at the participant’s home or at a mutually convenient location. An interview guide (Fig. 1) was utilised with questions concerning experience of the intervention, including the most and least useful aspects, and the factors influencing PABC. Interviews were digitally recorded and transcribed verbatim.

### Data analysis

Data were analysed by the first author using reflexive thematic analysis [15] to generate themes. Analysis included deductive and inductive approaches; whilst a codebook was not adopted, and categories were not pre-determined, it is recognised that the interview guide focused primarily on experiences of the intervention and therefore analysis was not entirely inductive. Nevertheless, data was ‘open-coded’ to best represent the perceptions and experiences (in relation to the research aims) as conveyed by participants [16]. Thematic reflexive analysis involved several steps including (i) *immersion* and the careful reading of transcripts, (ii) attaching codes to salient text segments and (iii) the identification of themes at a broader level and examining whether codes may be combined to form an overarching theme. During these processes, inductive analysis was used to generate themes grounded in the data. Although it is recognised that data interpretation may be influenced by the researcher’s prior knowledge, an attempt was made to be open to new findings that may conflict with theory and previous research findings [17, 18]. The final step involved reviewing themes, cross-checking for overlap and finally defining and classifying themes. The analysis offered is one interpretation of the data and other interpretations are possible. Nevertheless, we aim to offer a credible and trustworthy interpretation that accurately captures the data. For example, we provide ‘thick description’ via the use of extensive quotations so that the reader can evaluate the interpretation [19].

## Results

Twenty-three (70%) of invited participants completed an interview (mean age 65.8 ± 7.1 years). Table 1 displays participant demographics. The majority (*n*=18) were overweight (mean BMI of 28.8 ± 4.7). There were no significant differences in age, gender, cancer type, education, income or time since diagnosis between participants that entered the study compared to those who did not. Data analysis generated four main themes: commitment, accountability and monitoring, routine and Fitbit as health coach^1^*.* Table 2 provides an overview of themes and their content.

**Table 1:** Demographic characteristics of interview participants

		*N* (%)/*M* (*SD)*
Age (years)		65.8 (7.1)
Sex		
	Female	15 (65.2%)
	Male	8 (34.8%)
Marital status		
	Married	16 (69.6%)
	Divorced/separated	5 (21.7%)
	Single	1 (4.3%)
	Widowed	1 (4.3%)
Ethnicity		
	Caucasian	22 (95.7%)
	Indian	1 (4.3%)
Education		
	University degree	12 (52.2%)
	High school	6 (26.1%)
	Post-school training/qualification	5 (21.7%)
Household income (AUD)		
	≤ $30,000	4 (17.4%)
	$30,001–$52,000	7 (30.4%)
	$52,001–$104,000	5 (21.7%)
	$104,001–$156,000	5 (21.7%)
	$156,001–$208,000	1 (4.3%)
	$208,001–$260,000	1 (4.3%)
Smoking status		
	Non-smoker	17 (73.9%)
	Ex-smoker	6 (26.1%)
Comorbidities		
	Overweight	9 (31.1%)
	Obese	9 (31.1%)
	Hypertensive	7 (30.4%)
	Hypercholesterolemic	4 (17.4%)
	Diabetic	2 (8.7%)
Cancer type		
	Colorectal	16 (69.6%)
	Gynaecologic	7 (30.4%)
Treatment		
	Surgery only	10 (43.5%)
	Surgery with one adjuvant therapy	10 (43.5%)
	Surgery with two adjuvant therapies	3 (13.0%)
Time since diagnosis (years)		1.6 (0.9)

**Table 2:** Overview of themes and sub-themes

Theme	Sub-themes/codes	Brief description
Commitment	Prioritising physical activity Value of physical activity Perceiving external barriers	Commitment evidenced through prioritising and valuing PA was the foundational dimension that influenced PA engagement and behaviour change amongst participants. Those that assigned a higher value and priority to PA were more likely to establish a routine or schedule PA and be successful in PA behaviour change.
Accountability and monitoring	Accountability to the trial team Accountability to others General monitoring and support	Participants that reported a reliance on external accountability for motivation tended to be less successful in PA behaviour change. Most participants valued reviews/check ins but most of those that increased and maintained PA were not so focused on external accountability for motivation.
Routine	Incidental PA Scheduled PA Influence of retirement on routine***.***	Participants that established a PA routine by scheduling or planning exercise appeared more likely to engage in adequate MVPA and maintain PA. There was recognition of the importance of a routine for PA engagement and yet many that were retired referred to a lack of structure to their day and some were resistant to planning and structure since retirement.
Fitbit as health coach	Instilling awareness Prompts Self-monitoring and feedback Goal setting and review Unreliability of device Fitbit as a demotivator Technical challenges	The theme of ‘Fitbit as health coach’ summarises the finding that the Fitbit™ was perceived as the primary active ingredient to increase PA for most participants. The Fitbit was viewed as helpful in raising awareness of PA level and sedentary behaviour through objective feedback and as a self-monitoring tool to achieve goals and targets.

### Commitment

Commitment was underpinned by two sub-themes: prioritising PA and PA value. Participants who successfully increased MVPA were those who were committed to PA.

#### Prioritising PA

Commitment was expressed through prioritising health and PA: ‘I’m interested in my health…I’m invested’ (Graham, 67, > PA & M^2^); and ‘exercise had become the most important thing to me, so I had to reorganise my life’ (Felicity, 67, > PA and M). Those who successfully increased PA prioritised PA: ‘I don’t accept busy…you deserve to be able to book a time in your diary [to do PA] to arrange your day around it’ (Lyn, 68, > PA & M). Kevin linked exercise with longevity, which underlined his commitment to PA: ‘I wasn’t prepared to die at 60…if I’ve got to do an-hour of exercise a day then that’s easy enough’ (Kevin, 60, > PA and M). Participants less successful did not prioritise PA: ‘I put being a good Samaritan ahead of walking 10,000 steps’ (Oscar, 68, > PA & DNM), and ‘I’m not prepared to give up the things I am doing…They are more fulfilling than walking around the block a couple of times’ (Leah, 71, < PA). Those who did not prioritise PA adopted a more casual approach concerning exercise achievement: ‘I try have a little goal everyday of what I am going to do…sometimes I do it and sometimes I don’t’ (Katherine, 62, < PA) and ‘It was sort of between 6 to 8000-steps. I didn’t push it…even if I got to 3500 or 4000 still felt pretty good’ (Joe, 72, <PA and increased at T3). Priority underpinned motivation: those who prioritised PA were more committed whilst those who did not expressed lower motivation and more barriers. For example, ‘I understand that it should be a priority to me, but I’m not motivated…I became too busy, then I got sick and then it was too hot’ (Mary, aged 77).

#### Valuing PA

Participants who valued PA appeared to be more committed: ‘I didn’t need convincing about the importance of exercise…I want to be able to do the things I want to do at the age of 70 and 80 and into my 90s’ (Andrea, 64, >PA and M). Conversely, participants that were sceptical of the guidelines or doubted the importance of PA valued PA less and were less committed to PA. For example, ‘The aim was to get to 10,000-steps…I thought “well why? Am I going to feel any fitter?”’ (Joe, 72, < PA) and ‘I’ve adopted the attitude of I am going reasonably well. I am not a star pupil, but I’m not the lowest in the class…I did 6000 and that’s good’ (Oscar, 68, > PA and DNM).

### Accountability and monitoring

This theme included external accountability to the trial team for PA engagement, and more general monitoring which were perceived as helpful. Overall, participants that reported a reliance on external accountability for motivation tended to be less successful in PABC. For example, ‘It inspired me to get out because you’re accounting to someone about what you’re doing. I do need that’ (Leah, 71, <PA) and ‘I think some kind of monitoring…having some commitment and it’s an authority outside of you’ (Annette, 66, >PA but insufficiently active). The exception was Rebecca, who was one of the most successful participants yet almost entirely externally motivated: ‘I was motivated to do [PA] because it’s [trial co-ordinator’s] study…since I’ve finished the trial…there’s no motivation to keep exercising. It was the study keeping me motivated’ (Rebecca, 64, >PA and M).

Other participants reported that check-ins were motivational: ‘Maybe you’re more activated when someone is monitoring you’ (Christopher, 69, >PA and M) and ‘If I had a SMS or phone call to ask how it’s going or come have a review session that was a motivator’ (Graham, 67, >PA and M) or important for when encountering challenges: ‘I think the Fitbit, being aware of what I am doing, will be enough for me. Maybe if I knew there was a 3-monthly check-up would help me…when things go wrong like you get sick that’s when you need the motivation’ (Fiona, 67, >PA and M). Overall, most participants valued check-ins but most that successfully increased PA were not focused on external accountability for motivation.

### Routine

Routine included sub-themes of incidental PA and influence of retirement on routine*.* Participants that established a PA routine were more likely to engage in and maintain adequate MVPA: ‘I do feel that if you have a set routine you are going to make more of an effort to do it’ (Julie, 67, > PA and M); ‘We have got a pattern now of walking every night’ (Andrea, 64, >PA and M). Rebecca referred to the importance of planned PA to achieve the step goal: ‘Having that routine was really important…250 steps per hour doesn’t get you to 10000 so I usually have a bigger walk 20-25 minutes early in the morning and then again’ (Rebecca, 64, >PA and M).

#### Incidental PA

Those who engaged in more incidental PA were less likely to increase MVPA: ‘I should do [PA] on a regular basis… but it’s disciplining myself’ (Katherine, 62, <PA); ‘I never managed to set the time I was going to do it in advance, so the activity tended to be incidental’ (Renee, 59, < PA). Annette also referred to more incidental PA: ‘When it comes to stuff like this, I will just on the spot do something so I will just run up the stairs…exercise has never been a huge thing for me’ (Annette, 66, >PA but insufficiently active).

#### Influence of retirement on routine

Participants recognised the importance of routine, yet many who were retired referred to a lack of daily structure: ‘I need to have a time… but I find it hard now I’m retired to be as patterned in anything’ (Rachel, 76, >PA and DNM). Further, in several cases, there was a resistance to planning and structure since retirement: ‘Maybe it has something to do with not wanting to be regimented, like you are at work…regimented by deadlines and goals…so I object to being regimented as there is no fun in it’ (Oscar, 68, >PA and DNM).

### Fitbit as health coach

This theme summarises the finding that the Fitbit™ was the primary active ingredient to increase PA: ‘Well the Fitbit worked the best’ (Andrea, aged 64, >PA and M) and ‘The Fitbit was the motivator…it was kind of like everyday contact’ (Annette, 66 >PA and M) and Fitbit ‘was a motivator it was very powerful because it’s 24/7’ (Graham, 67, >PA and M). The Fitbit assisted in raising awareness of PA and sedentary behaviour through objective feedback and as a self-monitoring tool to achieve goals. The Fitbit did not work for all participants. Two participants that reduced MVPA had technical problems, for example ‘I couldn’t connect…the computer would say you need an update’ (Louisa, 76, <PA) and ‘My literacy is minimum…if I’d had a bit more training that would have helped’ (Mary, 77 <PA). Others referred to the unreliability of the Fitbit: ‘That was a major discourager…because I knew I could distort it by sweeping’ (Lyn, 68, >PA and M) or its inability to register PA:

If the Fitbit worked and I felt I was achieving something it might have encouraged me to do more exercise, but the Fitbit was a bigger disincentive…if you were doing short bursts, it would be very discouraging…it wasn’t recording even when I was making such an effort (Kath, 62, <PA)

Renee (59, <PA) recalled ‘I found that really frustrating…I would walk briskly (for) 7.5-minutes and it didn’t count…you get really annoyed when you do 9-minutes of vigorous activity, and it doesn’t count because it needs to be 10’. Fitbit as health coach contained three sub-themes: Prompts, self-monitoring and feedback, goal-setting and review.

#### Prompts

The Fitbit functioned as a prompt to decrease sedentary behaviour for most: ‘the bit most useful was the having to get up every 5-minutes…how easy it is to get 250-steps’ (Lyn, 68 >PA and M) and ‘Well the Fitbit was the tool to get your butt off the seat’ (Stephen, 44>PA and M).

#### Self-monitoring and feedback

The Fitbit provided self-monitoring and real-time feedback deemed motivational: ‘There were days where I wouldn’t look until 4pm and I would have only 3000 and I’d think I’ve got to go for an hour-long walk’ (Andrea, 64 >PA and M); ‘[The Fitbit] would say I had 8000 steps, so I would go around the block just to get 10000’ (Julie, 67 >PA and M). Participants who were more successful also reviewed their progress: ‘Fitbit would send you the weekly report and how you compared to last week. I looked at that and thought oh well this is what I’ve got to do’ (Julie, 67 >PA and M).

It is important to note that self-monitoring was the primary technique used by participants and that few engaged in or were willing to do formal action-planning despite action-planning being a core component of the intervention: ‘Action-planning doesn’t really work for me, I’m a list person…it has to something really simple and measurable…a tick on the calendar’ (Andrea, 64 >PA and M.). Most preferred to keep a diary as a self-monitoring tool: ‘I keep a diary…I would always write in my diary what I had done…walk lake, walk beach’ (Rachel, 76 >PA and DNM).

#### Goal-setting and review

For many, the Fitbit assisted with goal-setting: ‘I had a goal with the Fitbit I was able to keep going and maintain’ (Abigail, 67). Many set themselves a step goal ‘I just went with the 10000-steps’ (Rebecca, 64, >PA and M) or active minutes goal ‘I kept the goal of 150- minutes….my own goal was 210’ (Andrea, 64 >PA and M), and would review progress with the Fitbit: ‘I would do 3 or 4kms and it would only get about 4000-steps, and I thought yuck that’s not much so I would have to pick it up’ (Julie, 67, >PA and M) and ‘I always made the 150mins and nearly always met the 210’ (Andrea, 64, >PA and M). Those who increased MVPA assessed goal progress: ‘See yesterday was very light and I’ll compensate for that’ (Graham, 67 >PA and M). Conversely, those who were less successful set less challenging goals in order to feel a sense of achievement: ‘I set my goal low so I could always achieve, I set it at 7500-steps’ (Renee, 59 <PA) and ‘I looked at it and said I did 6000-steps today that’s terrific and if I only did 3500 that’s okay’ (Oscar, 68 >PA and DNM).

## Discussion

This study provides an in-depth understanding of the salient dimensions that influence PABC amongst colorectal and endometrial cancer survivors in addition to the successful ingredients of the intervention. Key themes generated explained the active ingredients of the intervention (i.e., Fitbit as health coach) and the dimensions associated with PABC more generally: commitment, accountability/monitoring and routine.

The study found clear differences in priorities between participants that successfully increased and maintained MVPA compared to those that did not. A commitment to PA appeared to be the foundation to successful PABC. Those that did not prioritise PA expressed lower motivation and presented more barriers to exercise. Similar findings were identified in a study on successful PA maintenance, where cancer survivors prioritised PA over other obligations [10]. Grimmett et al. (2020) also found that health benefits of PA were highly valued amongst gastrointestinal cancer survivors who had maintained PA following intervention [8]. In the present study, participants that deemed PA as essential for health were more successful in PABC, whereas those that were sceptical of the guidelines or doubted the importance of PA valued PA less and were less committed to PA. Similar findings concerning scepticism of health guidelines amongst cancer survivors have been recognised previously [20]. Consistent with previous research, low motivation and low priority typified those that did not increase MVPA during the intervention [8, 9, 20]. However, contrary to previous findings [8], a lack of enjoyment did not typify those that did not increase MVPA, nor did enjoyment play an obvious role in exercise maintenance. Previous research has similarly found that instrumental attitude, but not affective attitude, predicted PA intention in cancer survivors [21, 22] whilst other research supports relations between affective attitudes, and PA participation [23, 24].

Participants that developed a PA routine appeared more likely to increase MVPA consistent with previous research [8]. This is unsurprising, since those who are more committed to exercise are more likely to schedule PA. Scheduling exercise has been identified as a facilitator of PA engagement amongst cancer survivors [25]. Conversely, participants who engaged in more incidental PA were less likely to increase MVPA. This is a novel finding and indicates that a focus on accumulating steps is unlikely to be sufficient to achieve the PA guidelines.

A further novel finding was the resistance to structure and planning, related to retirement and a desire for less structure where formal scheduling was rejected as a reminder of employment. Although successful participants developed a PA routine, most did not engage in formal action-planning, despite it being a core intervention component. Instead, participants used a combination of goal-setting, self-monitoring, Fitbit-derived feedback and review, to evaluate progress and change behaviour accordingly. Self-monitoring was the primary technique used to sustain motivation and PA.

Reviews also support the role of self-monitoring for PABC [26–28] in addition to goal-setting [27, 28] and action-planning [26, 29, 30] in cancer survivors. In the present study, commitment led to goal-setting, which was kept in check through daily self-monitoring and review of behaviour. The Fitbit did not work for all participants; some experienced technical problems and others were demotivated due to its inability to accurately reflect users’ PA. Similar findings of cancer survivors rejecting wearables due to discrepancies between perceived PA and data provided by the device have been reported [9].

Finally, participants reliant on external accountability tended to be less successful in PABC. The desire for external accountability and monitoring to produce accountability in survivors has been reported elsewhere [9, 31]. Check-ins were valued, although most who were successful were not focused on external accountability for motivation.

### Study limitations

Our study recruited participants in Western Australia; therefore, findings may not be generalizable. The potential for selection bias and recall bias are further limitations. Strengths of the study include the high response rate, the focus on MVPA, lengthy interviews and capture of participants with varying PABC success.

## Conclusion

To our knowledge, this is the first study to comprehensively explore endometrial and CRC survivors’ experiences of a low-intensity intervention and to identify the salient dimensions that influenced PABC. Commitment was the foundational dimension that influenced PABC. Those who assigned a higher priority to PA were more likely to schedule PA and be successful. Those less successful lacked motivation and tended to engage in more incidental PA. Interventions that enhance commitment through increasing the value of PA would be worthwhile. Wearables hold promise in PA promotion and harnessing the technique of discrepancy between behaviour and goals is likely a valuable technique. Given the disappointment associated with devices failing to register short bouts of MVPA, future wearable would do well to ensure that all MVPA is captured.

### Supplementary information

ESM 1 (DOCX 18 kb)

## Footnotes

1 Overall, 12 (52%) increased MVPA and maintained PA at T3 and one increased MVPA during the intervention but did not maintain PA. Seven participants (30%) did not change their MVPA during the intervention and three (13%) reduced MVPA over the course of the intervention but remained physically active.

2 The identifiers following each quote include pseudonym, age, symbols (> or <) to denote increased or decreased MVPA (derived from the Actigraph GTX9 accelerometer) from T1 to T2, and M or DNM to denote maintained or did not maintain MVPA at T3 (24 weeks).
